# Circ_RUSC2 Sequesters miR-661 and Elevates TUSC2 Expression to Suppress Colorectal Cancer Progression

**DOI:** 10.3390/ijms26072937

**Published:** 2025-03-24

**Authors:** Yixin Shi, Dingru Li, Yunchao Xu, Yijun Guo, Jun Mao, Ying Lu

**Affiliations:** 1 Liaoning Laboratory of Cancer Genomics, Department of Cell Biology, College of Basic Medical Sciences, Dalian Medical University, Dalian 116044, China; 2 School of Biology and Biological Engineering, South China University of Technology, Guangzhou 510641, China; 3 Department of Psychology, Dalian Medical University, Dalian 116044, China; 4 Department of Medical Morphology Laboratory, College of Basic Medical Sciences, Dalian Medical University, Dalian 116044, China

**Keywords:** colorectal cancer, circ_RUSC2, microRNA-661, TUSC2, METTL3

## Abstract

Background: Despite advancements in diagnostic efficiency, colorectal cancer (CRC) remains a leading cause of cancer-related mortality, with increasing incidence rates. Circular RNA (circRNA) is a closed-loop, generally stable noncoding RNA that functions as a sponge for microRNAs in CRC. The purpose of this study was to investigate the function and underlying mechanism of circ_RUSC2, a new circRNA, in CRC. The expression levels of circ_RUSC2, miR-661, and TUSC2 were assessed using qRT-PCR, Western blot, and immunohistochemistry. Functional assays, including CCK-8, Transwell, and scratch wound healing, were performed to evaluate cell proliferation, migration, and invasion. RNA pull-down and actinomycin D assays were used to study RNA interactions and stability. In both CRC cells and tissues, miR-661 was markedly elevated, while circ_RUSC2 expression was considerably reduced. Poor differentiation, distant metastases, lymph node metastases, and an advanced stage were all strongly correlated with either miR-661 overexpression or circ_RUSC2 downregulation. circ_RUSC2 was more stable compared to its linear RUSC2 mRNA. CRC cell invasion, migration, and proliferation were suppressed by circ_RUSC2 ectopic expression; this inhibitory effect was restored by a miR-661 mimic. Circ_RUSC2 served as miR-661’s sponge. TUSC2 counteracted the effects of miR-661, which stimulated CRC cell proliferation, migration, and invasion. At the post-transcriptional level, miR-661 controlled the expression of TUSC2 in CRC cells. In comparison to the negative control, circ_RUSC2 expression was markedly reduced, and its half-life was shortened by methyltransferase-like 3 (METTL3) knockdown. Circ_RUSC2 is a stable cytoplasmic circRNA. Circ_RUSC2 inhibits CRC cell malignant phenotypes via the miR-661/TUSC2 axis. The onset and progression of CRC are linked to the downregulation of Circ_RUSC2. circ_RUSC2 might become more stable through N6-methyladenosine (m6A) methylation regulated by METTL3. According to our research, circ_RUSC2 might be a new biomarker and treatment target for CRC.

## 1. Introduction

According to global cancer statistics, colorectal cancer (CRC) ranks as the third most commonly diagnosed cancer worldwide and the second-leading cause of cancer-related mortality [1]. Crucially, China is going through a cancer shift, which is why the prevalence of CRC is rising quickly there. In 2020, the number of new cases in China was third for both sexes, and the fatality rate was second for women and fifth for men. The prognosis for people with CRC has improved due to recent advancements in therapy and multidisciplinary care. However, the number of deaths from CRC remains high, which may be as a result of the disease’s comparatively more prevalent late-stage presentation in China [2]. Therefore, it is critical to have a thorough understanding of the biology behind the onset and progression of CRC and to identify emerging biomarkers for treatment and assessment.

Noncoding RNAs (ncRNAs) that have a closed-loop structure are known as circular RNAs (circRNAs). Due to this unique structure, circRNAs are resistant to exonucleases and are more stable than linear mRNAs or long noncoding RNAs [3,4,5]. An increasing number of studies have shown that circRNAs contribute to the carcinogenesis of multiple cancers, including CRC, and may act as potential biomarkers based on their abundance and stability [6,7,8]. One of the well-known mechanisms by which circRNAs elicit their function is through serving as a microRNA (miRNA) sponge to prevent the miRNA-mediated cleavage of target mRNAs [9,10]. A series of circRNAs have been identified to exert function by absorbing miRNAs in CRC [11]. For instance, circTATDN3 stimulated CRC cell growth and the Warburg effect via sponging miR-511-5p and upregulating LDHA expression [6]. CircTBC1D22A played an anti-oncogenic role in CRC malignant progression through the regulation of ATG14 expression, which was mediated by sponging miR-1825 [12]. Circ_RUSC2 (also named hsa_circ_0002702), which originates from exon 2 of the RUN and SH3 domain-containing 2 (RUSC2) gene, has been reported to facilitate the vascular smooth muscle cell (VSMC) migration and proliferation by sponging miR-661 recently [13]; however, the role of circ_RUSC2 in CRC biology has not yet been clearly explained.

miRNAs, another class of ncRNAs, bind to the 3′ untranslated region (3′UTR) of target mRNAs, thereby modulating their translation efficiency or stability [14]. Sequence analysis revealed two potential binding sites for miR-661 within the circ_RUSC2 sequence. MiR-661 has been reported as a regulatory molecule that can control cell proliferation, invasion, and metastasis in a variety of neoplasms, such as non-small-cell lung carcinoma (NSCLC) [15,16,17], ovarian cancer [18], breast cancer [19], sinonasal squamous cell carcinoma [20], and pancreatic ductal adenocarcinoma (PDAC) [21]. Nevertheless, the roles of miR-661 in CRC remain poorly characterized. Tumor suppressor candidate 2 (TUSC2), also referred to as FUS1, is located on chromosome 3p21.3 [22]. Several recent studies have shown TUSC2 to elicit an anti-tumor effect [23,24].

In this study, we quantitatively assessed the expression levels of circ_RUSC2, miR-661, and TUSC2 mRNA in paired CRC tissues and adjacent non-tumor tissues and evaluated the correlation between their expressions and clinicopathological characteristics in CRC. Furthermore, we performed in vitro experiments to investigate the function of circ_RUSC2 in CRC cell proliferation, migration, and invasion and revealed the mechanism of circ_RUSC2 targeting the miR-661/TUSC2 axis. Finally, we investigated whether circ_RUSC2 stability was modulated by N6-methyladenosine (m6A) methylation.

## 2. Results

### 2.1. Circ_RUSC2 Is Notably Reduced in CRC Cells and Tissues, Correlating with Poor Clinicopathological Profiles

**Figure 1:**
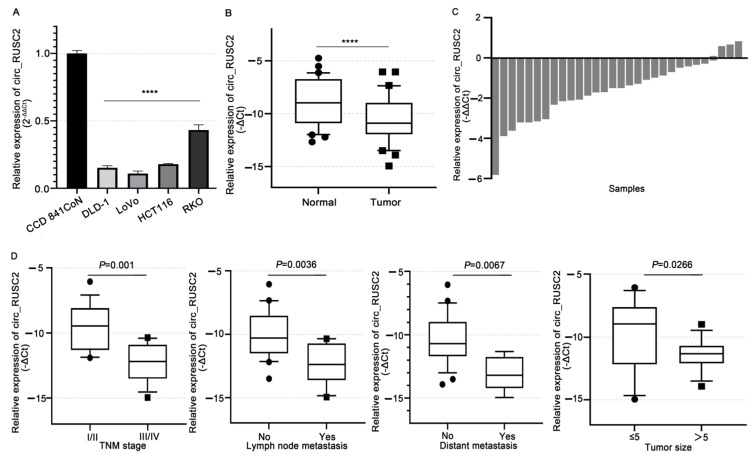
Circ_RUSC2 expression is decreased in CRC cells and tissues and is associated with poor clinicopathologic features. (**A**,**B**) Comparison of circ_RUSC2 expression in DLD-1, LoVo, HCT116, and RKO cells and CRC tissues, and CCD841 CoN cells, and normal adjacent colorectal tissues by qRT-PCR. (**C**) Relative expression level of circ_RUSC2 in CRC tissues and normal adjacent colorectal tissues of each case. (**D**) Analysis of clinicopathologic significance between relative expression of circ_RUSC2 and TNM stage, lymph node metastasis, distant metastasis, and tumor size. 18S rRNA was the internal control of circ_RUSC2. Data were indicated as mean ± SD from 3 independent experiments. **** *p* < 0.0001.

Firstly, we used qRT-PCR to detect the circ_RUSC2 level in CRC cell lines. Figure 1A illustrates that, in comparison to the normal colorectal epithelial cell line CCD 841CoN, the endogenous circ_RUSC2 expression was significantly downregulated in DLD-1, LoVo, HCT116, and RKO cells (*p* < 0.0001). DLD-1 and LoVo cells exhibited the most pronounced reduction, and therefore, the two mentioned lines were selected for the next in vitro experiments.

Next, in a group of 31 matched CRC tissues, we evaluated the clinicopathological significance of circ_RUSC2 expression. Compared to the adjacent healthy colorectal tissues, we observed a substantial decrease in circ_RUSC2 expression (*p* < 0.0001, Figure 1B). Approximately 83.87% (26/31) of tumor tissues exhibited lower circ_RUSC2 levels compared to normal tissues (Figure 1C). Moreover, circ_RUSC2 levels were markedly lower in advanced stages (III–IV) than in early stages (I–II) (*p* < 0.0001) and showed an inverse correlation with tumor size, distant metastasis (*p* < 0.05), lymphatic metastasis *(p* < 0.001), and poor-to-moderate differentiation (*p* < 0.01, Figure 1D and Table S1) of CRC; in contrast, these levels were not associated with age (*p* = 0.0763), gender (*p* = 0.512) (*p* = 0.2879), or other factors (Supplementary Table S1 and Figure S1). These findings indicated that circ_RUSC2 expression is reduced in CRC cells and tissues, and the decreased circ_RUSC2 expression is related to advanced stage, lymphatic and distant metastases, and poor-to-moderate differentiation.

### 2.2. Circ_RUSC2 Is a Stable Cytoplasmic circRNA in CRC Cells

**Figure 2:**
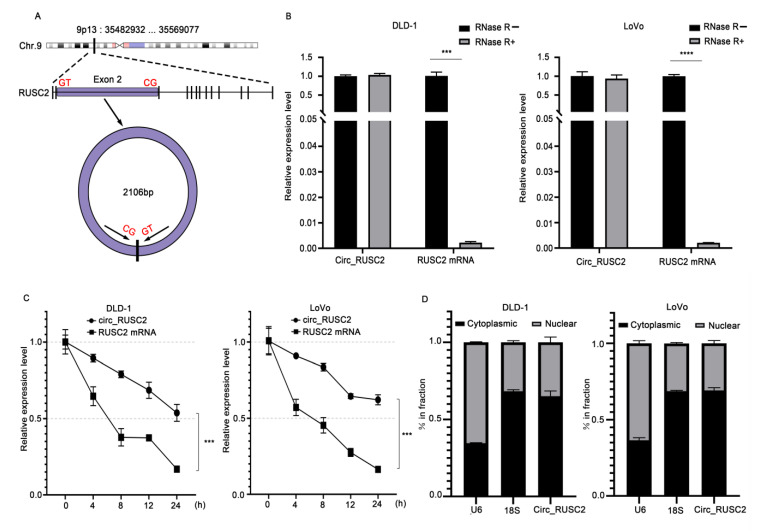
Circ_RUSC2 is a stable cytoplasmic circRNA in CRC cells. (**A**) Schematic diagram of circ_RUSC2 formation from exon 2 of the RUSC2 gene at chromosome 9p13. (**B**) Relative expression of circ_RUSC2 and linear RUSC2 mRNA by qRT-PCR after RNase R treatment on DLD-1 and LoVo cells. The expression level of circ_RUSC2 or linear RUSC2 mRNA without RNase R treatment was set as 1. (**C**) Relative expression of circ_RUSC2 and linear RUSC2 mRNA by qRT-PCR after actinomycin D treatment on DLD-1 and LoVo cells every 4 h for 24 h. The expression level of circ_RUSC2 or linear RUSC2 mRNA at 0 h was set as 1. GAPDH was the internal control of linear RUSC2 mRNA. (**D**) Percentage of U6, 18S rRNA, and circ_RUSC2 in the cytoplasmic and nuclear fraction. U6 was a nuclear RNA marker, and 18S rRNA was a cytoplasmic RNA marker. Data were shown as mean ± SD from 3 independent experiments. *** *p* < 0.001, **** *p* < 0.0001.

According to the annotation of circBase, circ_RUSC2 is a 2106 bp circular transcript originating from exon 2 of the RUSC2 gene located at chromosome 9p13 (Figure 2A). CircRNAs possess a greater stability compared to linear mRNAs because of the absence of 3′ and 5′ ends. The data of RNase R assay demonstrated that the expression of RUSC2 mRNA decreased substantially in DLD-1 (*p* < 0.001) and LoVo cells (*p* < 0.0001) relative to that without RNase R treatment (Figure 2B). However, following RNase R treatment, it was observed that circ_RUSC2 expression in these two cell lines was not obviously altered (Figure 2B), indicating that circ_RUSC2 is more resistant to RNase R degradation than linear RUSC2 mRNA. After actinomycin D treatment, we also evaluated the half-lives for the circ_RUSC2 transcript and linear RUSC2 mRNA transcript. In both cell lines, the half-life of circ_RUSC2 surpassed 24 h, whereas that of linear RUSC2 mRNA was roughly 6 to 8 h (Figure 2C). These data demonstrated that the form of circ_RUSC2 is steadier than the linear RUSC2 mRNA.

Exon-derived circRNAs are predominantly localized in the cytoplasm [25]. To determine the subcellular distribution of circ_RUSC2, we analyzed its expression levels in the nuclear and cytoplasmic fractions of DLD-1 and LoVo cells. Our results revealed that the proportion of circ_RUSC2 in the cytoplasm was comparable to that of 18S rRNA, but significantly higher than that of U6 (Figure 2D). This suggests that circ_RUSC2 is primarily localized in the cytoplasm of CRC cells. Together, these observations indicate that circ_RUSC2 is a stable circRNA with a cytoplasmic preference in CRC cells.

### 2.3. Circ_RUSC2 Sponges miR-661 as a ceRNA

**Figure 3:**
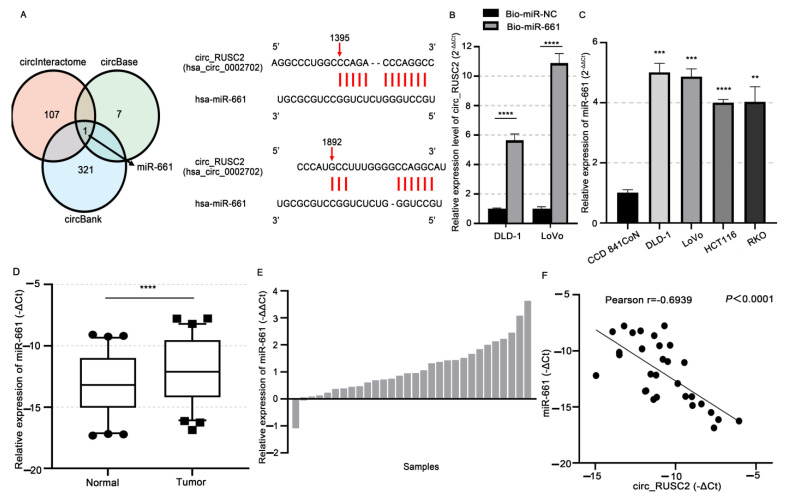
Circ_RUSC2 binds with miR-661 as a ceRNA in CRC. (**A**) Predicted circ_RUSC2-bound miRNA by databases and the binding sites between circ_RUSC2 and miR-661. (**B**) Enrichment of circ_RUSC2 by biotin-labeled miR-661 mimic. The biotin-miR-NC group was the negative control. (**C**) Comparison of miR-661 expression in DLD-1, LoVo, HCT116, and RKO cells and CCD841 CoN cells. (**D**) Comparison of miR-661 expression in the CRC tissues and paired normal colorectal tissues. (**E**) Relative expression level of miR-661 in CRC tissues and normal adjacent colorectal tissues of each case. (**F**) Spearman correlation analysis of circ_RUSC2 and miR-661 expression in the CRC cohort. Data are shown as mean ± SD from 3 independent experiments. ** *p* < 0.01, *** *p* < 0.001, **** *p* < 0.0001.

Based on the above results, we attempted to determine whether circ_RUSC2 could sponge miRNAs. We applied the bioinformatic analysis tools CircBank, CircInteractome, and CircBase to predict the associated miRNAs binding with circ_RUSC2. As shown in Figure 3A, these three databases showed one overlapping miRNA, i.e., miR-661, and two binding sites existed between circ_RUSC2 and miR-661. To confirm whether circ_RUSC2 could sponge miR-661 as a ceRNA, we conducted a biotin-coupled miRNA pull-down assay in DLD-1 and LoVo cells. The results showed that the expression level of circ_RUSC2 was significantly increased in the Bio-miR-661 group compared to that in the Bio-miR-NC group (*p* < 0.0001, Figure 3B), indicating that circ_RUSC2 is significantly pulled down and enriched by the biotin-labeled miR-661 mimic.

To further investigate the potential regulatory interaction between miR-661 and circ_RUSC2, we assessed the endogenous expression levels of miR-661 in CRC cell lines. As illustrated in Figure 3C, the miR-661 level was significantly elevated in DLD-1, LoVo, HCT116, and RKO cells compared to CCD 841CoN cells (*p* < 0.01). Furthermore, miR-661 expression was markedly higher in CRC tissues than in adjacent normal colorectal tissues (*p* < 0.0001, Figure 3D). The analysis of clinical samples revealed that 93.55% (29/31) of cases exhibited increased miR-661 levels in tumor tissues compared to normal tissues (Figure 3E). Spearman correlation analysis demonstrated a significant inverse correlation between miR-661 and circ_RUSC2 expression in CRC tissues (r = −0.6939, *p* < 0.0001, Figure 3F). Supporting this finding, a recent study identified miR-661 as a direct target of circ_RUSC2 in vascular smooth muscle cells (VSMCs) using luciferase reporter assays [16]. Collectively, these findings suggest that circ_RUSC2 may function as a competing endogenous RNA (ceRNA) by sequestering miR-661, thereby modulating its activity in CRC.

### 2.4. Circ_RUSC2 Inhibits the Proliferative, Migratory, and Invasive Behaviors of CRC Cells via Sponging miR-661

**Figure 4:**
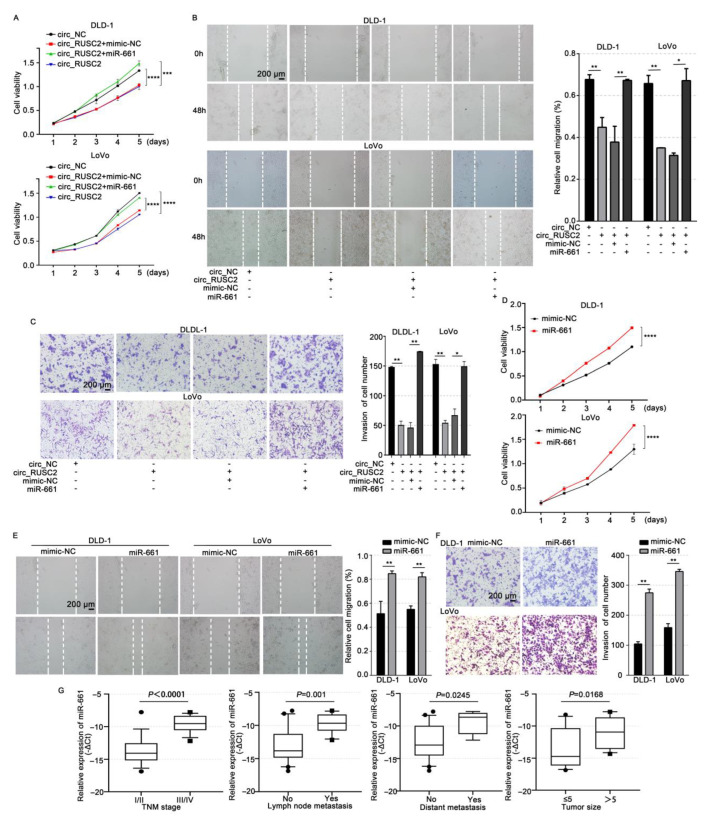
Circ_RUSC2 suppresses the capacities of CRC cell proliferation, migration, and invasion via sponging an oncomiR, miR-661. (**A**–**C**) DLD-1 and LoVo cells were transfected with circ_NC or circ_RUSC2, and mimic-NC or miR-661 mimic. Cell viability (**A**), migratory (**B**), and invasive (**C**) abilities were determined by CCK-8, and scratch wound healing and Transwell chamber assay with Matrigel. (**D**–**F**) DLD-1 and LoVo cells were transfected with mimic-NC or miR-661 mimic. Cell viability (**D**), migratory (**E**), and invasive (**F**) abilities were determined by CCK-8, scratch wound healing, and Transwell chamber assay with Matrigel. (**G**) Correlation of miR-661 expression with TNM stage, lymph node metastasis, distant metastasis, and tumor size. Data are shown as mean ± SD from 3 independent experiments. * *p* < 0.05, ** *p* < 0.01, *** *p* < 0.001, **** *p* < 0.0001.

To investigate the impact of circ_RUSC2 on CRC cells, we transfected the circ_RUSC2 plasmid into DLD-1 and LoVo cells, and qRT-PCR was used to confirm the transfection efficiency (Supplementary Figure S2). CCK-8 and scratch wound healing experiments displayed that the overexpression of circ_RUSC2 suppressed cell proliferative (*p* < 0.001 and 0.0001, respectively) and migratory (*p* < 0.01) abilities compared to the circ_NC group (Figure 4A,B). Figure 4 shows that the circ_RUSC2 group had considerably fewer cells piercing the membrane, according to the Transwell invasion test (*p* < 0.001 and 0.01, respectively). These results demonstrate that circ_RUSC2 suppresses CRC cell invasion, migration, and proliferation.

To determine whether the inhibitory effect of circ_RUSC2 is related to the targeting of miR-661, we co-transected the circ_RUSC2 plasmid and miR-661 mimic into DLD-1 and LoVo cells, and the co-transfection of circ_RUSC2 plasmid and mimic-NC was the negative control. The CCK-8 experiment displayed that the ectopic level of miR-661 due to transient transfection rescued the inhibitory effect of circ_RUSC2 overexpression on cell growth (*p* < 0.001 and 0.0001, respectively) in both CRC cells and tissues (Figure 4A), and the capacities of migration (*p* < 0.05 and 0.01, respectively) and invasion (*p* < 0.05) were also restored (Figure 4B,C). Thus, circ_RUSC2 has a tumor-suppressive role in CRC by inhibiting growth, invasion, and migration.

We further investigated the role of miR-661 in CRC. Functional assays revealed that the overexpression of miR-661 significantly enhanced the proliferation (*p* < 0.001 and *p* < 0.0001), migration (*p* < 0.01), and invasion (*p* < 0.01) capabilities of CRC cells compared to the mimic-NC control group (Figure 4D–F). Clinically, higher miR-661 activity was linked to the advanced stage (*p* < 0.0001), lymphatic metastasis (*p* = 0.001), distant metastasis (*p* = 0.0245), and poor-to-moderate differentiation (*p* = 0.0168, Figure 4G). These results suggest that miR-661 may function as an oncomiR in CRC.

### 2.5. TUSC2 Is Targeted by miR-661 in CRC Cells

**Figure 5:**
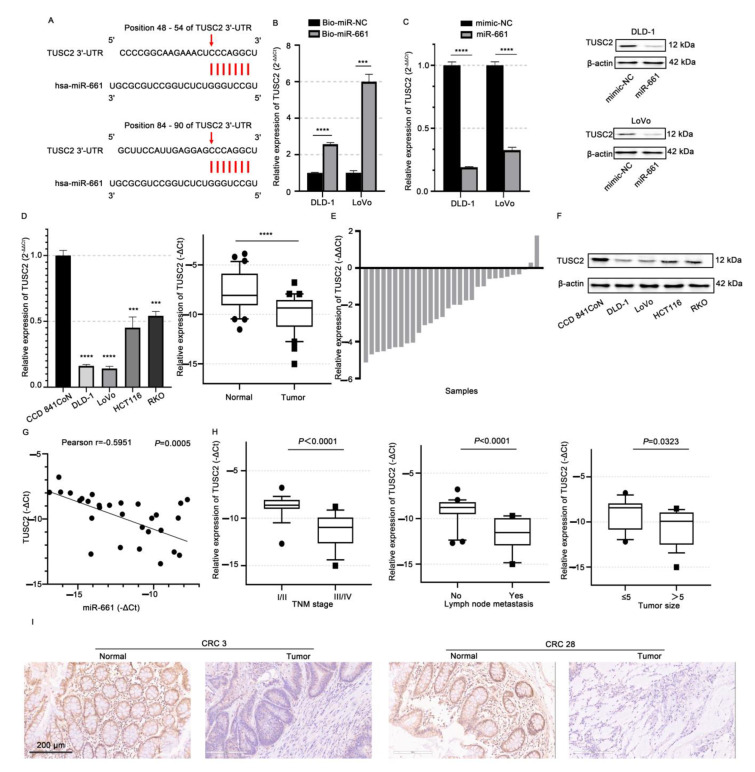
miR-661 negatively regulates TUSC2 in CRC cells. (**A**) Predicted binding site between miR-661 and 3’UTR of TUSC2 mRNA. (**B**) Enrichment of TUSC2 mRNA by biotin-labeled miR-661 probe pull-down. GAPDH was the internal control. (**C**) Relative expressions of TUSC2 mRNA and protein in DLD-1 and LoVo cells transfected with miR-661 or mimic-NC, and miR-661 inhibitor or inhibitor-NC. (**D**) Relative expression of TUSC2 mRNA in DLD-1, LoVo, HCT116, and RKO cells and CRC tissues, and CCD841 CoN cells, and normal adjacent colorectal tissues by qRT-PCR. (**E**) Comparison of TUSC2 mRNA between tumor tissues and paired adjacent normal intestinal tissues in each case. (**F**) TUSC2 protein expression in the CRC cell lines and CCD841 CoN cells by Western blot. β-actin was the internal control. (**G**) Association of miR-661 and TUSC2 mRNA expressions by Spearman correlation analysis. (**H**) Clinicopathologic significance analysis of TUSC2 mRNA expression in CRC patients. Data are shown as mean ± SD from 3 independent experiments. (**I**) Immunohistochemistry showed that the expression level of TUSC2 was decreased in tumor tissues compared with normal tissue *** *p* < 0.001, **** *p* < 0.0001.

A circRNA acting as a ceRNA facilitates its function through releasing the targeted mRNAs of miRNAs. Since circ_RUSC2 inhibited the malignant behaviors of CRC, here, we focused on the putative targets with tumor-suppressive function in the database. TUSC2 plays a tumor-suppressive role in a range of human malignancies [23,24]. Intriguingly, miR-661-binding sites were predicted to be present in the 3’UTR of TUSC2 mRNA using the bioinformatic analysis of TargetScan (Figure 5A). Using a luciferase reporter experiment, Liu et al. [26] recently revealed that miR-661 directly targets TUSC2 at the post-transcriptional stage in esophageal cancer cells.

To testify the regulative link between miR-661 and TUSC2, first, we conducted the RNA pull-down assay in DLD-1 and LoVo cells. When compared to the negative control, biotin-labeled miR-661 probe treatment significantly increased the expression of TUSC2 mRNA (*p* < 0.001, Figure 5B). Next, we detected the levels of TUSC2 mRNA and protein in DLD-1 and LoVo cells after transfection with the miR-661 mimic. The transfection efficiency of miR-661 was confirmed (Supplementary Figure S3). Compared with the mimic-NC group, miR-661 significantly decreased the expressions of TUSC2 mRNA (*p* < 0.01 and 0.0001, respectively) and protein in both CRC cells and tissues (Figure 5C). In CRC cells, miR-661 appears to negatively modulate TUSC2 mRNA expression post-transcriptionally.

We further detected the endogenous TUSC2 mRNA expressions in CRC cells and tissues. The TUSC2 mRNA level was significantly reduced in both CRC cells and tissues (*p* < 0.0001, Figure 5D). About 93.55% (29/31) of cases showed a decreased TUSC2 mRNA level in CRC tissues (Figure 5E). Consistently, the TUSC2 protein expression was decreased in CRC cells (Figure 5F). The expression levels of miR-661 and TUSC2 mRNA showed an inverse correlation in CRC tissues (*r* = −0.5951, *p* = 0.0005, Figure 5G), further confirming that miR-661 regulates TUSC2. In addition, the reduced TUSC2 mRNA levels were significantly associated with advanced tumor stages (*p* < 0.0001), the presence of lymph node metastasis (*p* < 0.0001), and a poorer histological differentiation (*p* < 0.05, Figure 5H). Immunohistochemical analysis further confirmed that the TUSC2 protein expression was markedly higher in normal intestinal epithelial tissues compared to tumor tissues (Figure 5I).

### 2.6. Circ_RUSC2 Upregulates TUSC2 Protein Expression of CRC Cells Through miR-661 Sponging

**Figure 6:**
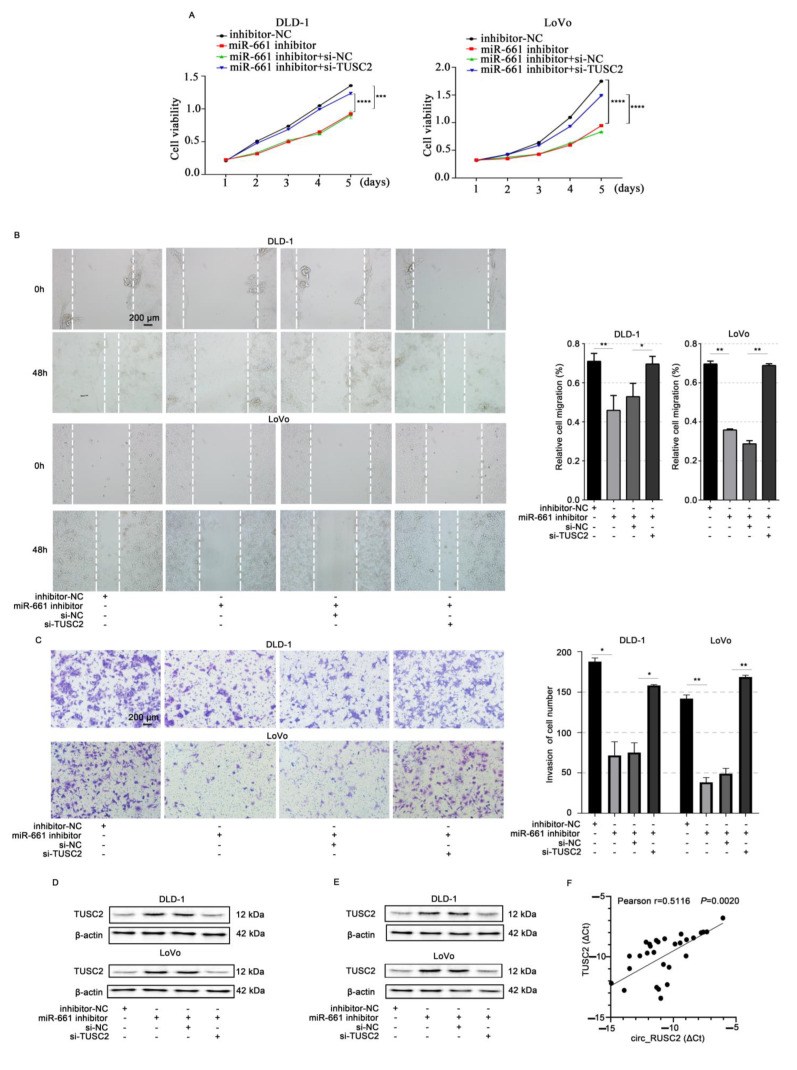
Circ_RUSC2 increases TUSC2 protein expression through sponging miR-661 in CRC cells. (**A**–**D**) DLD-1 and LoVo cells were transfected with inhibitor-NC or miR-661 inhibitor, and si-NC or si-TUSC2. Cell viability (**A**), migratory (**B**), and invasive (**C**) abilities were determined by CCK-8, scratch wound healing, and Transwell chamber assay with Matrigel, and TUSC2 protein was detected by Western blot (**D**). (**E**) DLD-1 and LoVo cells were transfected with circ_NC or circ_RUSC2, and mimic-NC or miR-661 mimic. TUSC2 protein was examined by Western blot. (**F**) Spearman correlation analysis of circ_RUSC2 and TUSC2 mRNA expressions in the CRC cohort. Data are shown as mean ± SD from 3 independent experiments. * *p* < 0.05, ** *p* < 0.01, *** *p* < 0.001, **** *p* < 0.0001.

To explore the mechanistic role of circ_RUSC2 in regulating malignant behaviors of CRC cells, DLD-1 and LoVo cells were transfected with a miR-661 inhibitor and si-TUSC2. The findings demonstrated that the inhibition of miR-661 significantly suppressed CRC proliferative (*p* < 0.001), migrative (*p* < 0.01), and invasive cell capacities (*p* < 0.01, Figure 6A–C). However, these inhibitory effects were antagonized by TUSC2 silencing (Figure 6A–C). Moreover, the downregulation of miR-661 notably increased the TUSC2 protein level compared to the inhibitor-NC group (Figure 6D). The co-transfection of miR-661 inhibitor and si-TUSC2 decreased the TUSC2 protein expression in contrast to that in the 661in+si-NC group while showing a similar level of TUSC2 protein to that in the inhibitor-NC group (Figure 6D). These results indicate that miR-661 downregulation inhibits CRC cell proliferation, migration, and invasion, possibly through the upregulation of TUSC2.

Additionally, Western blot data indicated that the TUSC2 protein level was increased by circ_RUSC2 overexpression. However, the miR-661 mimic attenuated the impact of circ_RUSC2 on the TUSC2 protein level in DLD-1 and LoVo cells (Figure 6E). Circ_RUSC2 and TUSC2 mRNA expression exhibited a positive correlation in CRC tissues (*r* = 0.5116, *p* = 0.002, Figure 6F). Together, these findings suggested that circ_RUSC2 increases the TUSC2 protein expression by acting as a sponge for miR-661.

### 2.7. N6-Methyladenosine (m6A) Modification Stably Increases the circ_RUSC2 Expression and Is Possibly Mediated by METTL3

**Figure 7:**
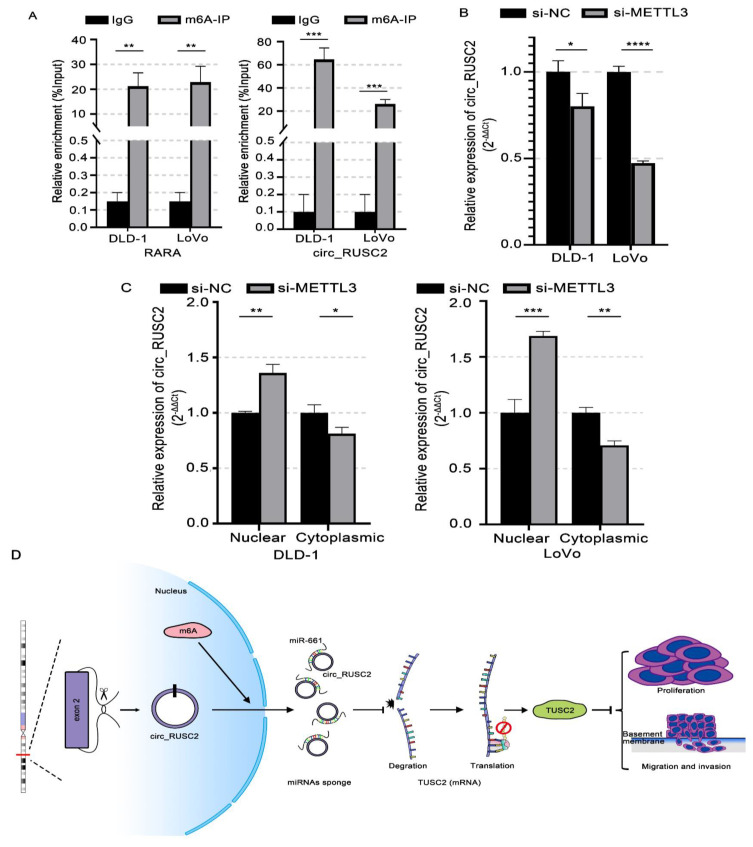
METTL3-mediated m6A modification increases the stability of circ_RUSC2 expression. (**A**) The MeRIP experiment proved the existence of m6A methylation site of circ_RUSC2. (**B**) Relative expression of circ_RUSC2 after knocking down METTL3. (**C**) The expression difference of circ_RUSC2 in the nucleus and cytoplasm after knocking down METTL3. (**D**) Mechanism of circ_RUSC2. Data are shown as mean ± SD from 3 independent experiments. * *p* < 0.05, ** *p* < 0.01, *** *p* < 0.001, **** *p* < 0.0001.

Recent studies have highlighted the critical role of m6A RNA methylation in colorectal cancer (CRC) progression and patient survival, as revealed by the high-throughput sequencing analyses of the m6A methylome in CRC patients [27]. Among the key regulators of m6A modification, methyltransferase-like 3 (METTL3) acts as a core “writer” enzyme, and its involvement in CRC carcinogenesis has been increasingly recognized [28,29]. The Me-RIP showed that circ_RUSC2 has a m6A methylation modification site (Figure 7A). Furthermore, we determined whether circ_RUSC2 can be affected by METTL3. The analysis of qRT-PCR indicated that METTL3 knockdown significantly decreased the expression of circ_RUSC2 (Figure 7B), indicating that circ_RUSC2 might be affected by METTL3. Furthermore, we employed actinomycin D treatment to assess the stability of circ_RUSC2 in the context of METTL3 silencing. Strikingly, METTL3 depletion led to a marked reduction in the half-life of circ_RUSC2 compared to the negative control (*p* < 0.01, Figure 7C), suggesting that METTL3-mediated m6A methylation enhances circ_RUSC2 stability.

## 3. Discussion

Differentially expressed circRNAs contribute to the CRC biological processes such as cell proliferation, apoptosis, migration, invasion, metastasis, and drug resistance. In this study, our findings revealed that circ_RUSC2 expression was markedly downregulated in CRC tissues and cells. Importantly, this downregulation was associated with more aggressive disease features, including advanced clinical stages, lymphatic and distant metastases, and poor tumor differentiation. Circ_RUSC2 was shown to suppress CRC cell invasion, proliferation, and migration, potentially through its interaction with miR-661, leading to the upregulation of TUSC2 expression. Additionally, the stability of circ_RUSC2 was significantly reduced upon METTL3 knockdown. To the best of our knowledge, this study is the first to uncover the tumor-suppressive role of circ_RUSC2 in CRC, elucidate its molecular mechanism involving the miR-661/TUSC2 axis, and identify METTL3 as a key upstream regulator of circ_RUSC2 stability.

Previous studies have consistently demonstrated the downregulation of circRNAs in CRC. Bachmayr-Heyda et al. [30] initially reported a global reduction in circRNAs in CRC tissues compared to normal mucosa. Subsequent studies further supported this trend: Wang et al. [31] identified hsa_circ_001988 as a downregulated circRNA in CRC, with its reduction linked to perineural invasion and poor differentiation; Zhuo et al. [32] observed a decreased expression of circRNA0003906 in CRC cell lines and tissues, correlating with poor differentiation and lymphatic metastasis; and hsa_circ_0000567 was found to be significantly reduced in CRC, with its low expression negatively associated with tumor size, TNM stage, distant metastasis, and lymph node metastasis [33]. Additionally, circFBXW4 and circ_0087851 were found to be downregulated in CRC tissues and linked to unfavorable clinicopathological features [34,35]. In line with these findings, our study revealed that circ_RUSC2 expression was obviously decreased in CRC tissues and cell lines, and its downregulation was related to poor differentiation, lymphatic, distant metastasis, and advanced tumor stages. Thus, circ_RUSC2 might be a suppressor of CRC development and progression. Although multiple circRNAs have been implicated in CRC pathogenesis, the unique expression pattern and functional role of circ_RUSC2 suggest that it plays a distinct role in CRC progression. Future studies will further investigate the unique contribution of circ_RUSC2 to CRC biology and its potential as a therapeutic target by comparing its expression and function with those of other CRC-associated circRNAs. Sun et al. [13] recently found that circ_RUSC2 promoted VSMC proliferation and migration. However, the function of circ_RUSC2 in tumors has not yet been reported. Our results revealed its expression and clinicopathologic significance in CRC.

The miRNA sponge has become the most well-known mechanism of circRNA function since 2013 [9,10]. Our data indicated that circ_RUSC2 engages with miR-661 through a direct binding mechanism in CRC cells. MiR-661 expression was high in both CRC cells and tissues, and a higher level of miR-661 expression was associated with poor clinicopathologic features of CRC patients. MiR-661 enhanced the proliferation, migration, and invasion of CRC cells. MiR-661’s oncogenic role has been reported in NSCLC [15,17], ovarian cancer [18], breast cancer [19], sinonasal squamous cell carcinoma [20], and PDAC [21]. The level of blood miR-661 was upregulated in HCC and NSCLC patients [16,36]. However, there are some contradictions about its role in cancer. To the best of our knowledge, this is the first report of miR-661 acting as an oncomiR in CRC.

CircRNAs elicit their effects through sponging miRNAs and subsequently relieve the miRNA target genes [10]. TUSC2 emerges as a pivotal tumor suppressor, with its decreased expression commonly observed in various cancers, including lung cancer [37], malignant mesothelioma [38], soft-tissue sarcoma [39], high-grade human glioma [40], and thyroid cancer [41]. Several miRNAs have been found to target TUSC2 mRNA.

Importantly, miR-661 stands out as a direct mediator regulating TUSC2 mRNA, confirmed through luciferase reporter assays in esophageal cancer EC109 cells [26]. Our investigations revealed a novel insight into CRC cells, where miR-661 negatively modulated TUSC2 mRNA expression post-transcriptionally. Notably, the suppression of CRC cell proliferation, invasion, and migration achieved through miR-661 downregulation was reversed upon the silencing of TUSC2, suggesting that miR-661 exerts oncogenic effects, potentially by targeting TUSC2. The TUSC2 protein consists of 110 amino acids and predominately resides in the mitochondria [42,43]. Current studies have demonstrated that TUSC2 might play a tumor-suppressing role through inducing apoptosis, arresting the G1 cell cycle, and inhibiting proliferation-related kinases, e.g., EGFR, mTOR, and AKT [24]. A recent study reported TUSC2’s tumor-suppressing activity in thyroid cancer via SMAC/DIABLO and CYTOCHROME C protein [41]. Combined with our experimental results, we hypothesized that TUSC2 repressed the malignant phenotypes of CRC cells, possibly by the induction of cell cycle arrest and apoptotic proteins and the inhibition of proliferation-related kinases, but further elucidation of this hypothesis is necessary to fully comprehend the underlying mechanisms.

Compared with most research focusing on the downstream mechanism of circRNA affecting tumorigenesis and progression, few studies concentrate on the upstream regulation of circRNAs [44]. m6A has been reported to participate in the splicing, transportation, stabilization, and degradation of mRNAs and ncRNAs in carcinogenesis [45]. A recent study showed that by upregulating MAP3K9 and sponging miR-186-3p, the METTL14’s m6A alteration of circUGGT2 has been shown to increase GC carcinogenesis and DDP resistance [46]. Our results showed that METTL3 knockdown induced a significant decrease in the circ_RUSC2 level. We also found that circ_RUSC2 stability was influenced by METTL3 knockdown. Li et al. [47] found an increased m6A level of circSLCO1B3 in HuCCT1 cells. The circSLCO1B3 level was decreased by METTL3 and YTHDC1 knockdown, and the m6A modification can stabilize circSLCO1B3. Our findings align with the recent findings of Wu et al. [48], suggesting that METTL3 plays a crucial role in maintaining the cytoplasmic stability of circCUX1 in head and neck tumor cell lines. However, more data are needed to reveal the regulatory interplay between circ_RUSC2 and METTL3 and the possible binding motif.

The significant downregulation of circ_RUSC2 in CRC tissues and its correlation with advanced clinicopathological features suggest its potential as a diagnostic and prognostic marker. Future studies should further evaluate the diagnostic accuracy of circ_RUSC2 and compare it with existing markers such as CEA and CA19-9. Additionally, determining whether circ_RUSC2 affects the sensitivity of CRC cells to commonly used chemotherapy drugs (such as 5-FU and oxaliplatin) and targeted therapy drugs (such as cetuximab) will provide a reliable basis for the clinical diagnosis and treatment of CRC.

## 4. Materials and Methods

### 4.1. Cell Culture

The human colorectal carcinoma cell lines HCT116 and RKO were sourced from the Cell Bank of the Chinese Academy of Sciences (Shanghai, China), while the DLD-1 cell line was acquired from the BeNa Culture Collection (Beijing, China). The normal human colon epithelial cell line CCD 841 CoN and CRC cell line LoVo were obtained from iCell Bioscience Inc (Shanghai, China). LoVo, CCD 841 CoN, and HCT116 cells were propagated in RPMI-1640 medium (Gibco, Shanghai, China), whereas RKO and DLD-1 cells were cultivated in DMEM medium (Gibco, Shanghai, China). Both media were enriched with 10% fetal bovine serum (FBS; Biological Industries, Kibbutz Beit Haemek, Israel) and 1% penicillin/streptomycin (Gibco, Shanghai, China). All cell lines were maintained in a controlled environment with 5% CO_2_ at 37 °C under humidified conditions.

### 4.2. Tissue Samples

A total of 31 paired samples of CRC and adjacent non-tumor tissues, confirmed by microscopic examination, were acquired from Shanghai Outdo Biotech Company (Shanghai, China). The utilization of these human tissue samples complied with all applicable ethical guidelines and regulations, and the study protocols were reviewed and approved by the Ethics Committee of Shanghai Outdo Biotech Company.

### 4.3. Cell Transfection

The circ_RUSC2 overexpression plasmid (circ_RUSC2), small interfering RNA (siRNA) targeting TUSC2 (si-TUSC2), siRNA targeting methyltransferase-like 3 (si-METTL3), and their respective negative controls (circ_NC and si-NC), as well as the miR-661 mimic (miR-661), miR-661 inhibitor, and their respective negative controls (mimic-NC and inhibitor-NC), were procured from GenePharma (Suzhou, China). The sequences for the circRNA, siRNA, miRNA, and miRNA inhibitor are provided in Supplementary Table S1. LoVo and DLD-1 cells were seeded in 6-well plates at a density of 2 × 10^5^ cells per well. After 24 h, the transfection of circ_RUSC2, miR-661 mimic, miR-661 inhibitor, si-METTL3, or their respective negative controls was performed using Lipofectamine 2000 (Invitrogen, Carlsbad, CA, USA). For the co-transfection of the circ_RUSC2 plasmid and miR-661 mimic (circ_RUSC2+miR-661) or miR-661 inhibitor and si-TUSC2 (661-inhibitor+ si-TUSC2), the quantity of each RNA molecule per group was reduced to half.

### 4.4. RNA Isolation and Quantitative Reverse-Transcription PCR (qRT-PCR)

Total RNA was extracted from transfected cells and freshly frozen tissues using TRIzol reagent (Invitrogen, Carlsbad, CA, USA) following the manufacturer’s instructions.

For the quantification of circ_RUSC2, miR-661, RUSC2, and TUSC2 mRNAs, cDNA synthesis was carried out using the Evo M-MLV RT Kit with gDNA Clean for qPCR II (AG11711, Accurate Biotechnology, Changsha, China). Real-time PCR was subsequently performed using the SYBR Green Premix Pro Taq HS qPCR Kit (AG11701, Accurate Biotechnology, Changsha, China) on an Agilent MX3000P system. The levels of circ_RUSC2 were normalized to 18S rRNA, while GAPDH served as the internal control for RUSC2 and TUSC2 mRNAs. For miR-661, U6 was used as the normalization reference. The relative levels of circ_RUSC2, miR-661, RUSC2, and TUSC2 mRNAs were determined using the 2^−ΔΔCt^ method in CRC cell lines [49]. In tissue samples, the expression levels of circ_RUSC2, miR-661, and TUSC2 mRNA were calculated using the -ΔCt method [50]. The primers for circ_RUSC2 were obtained from GenePharma (Suzhou, China), and the remaining primers were sourced from Invitrogen (Carlsbad, CA, USA). The primer sequences are detailed in Supplementary Table S2.

### 4.5. Actinomycin D and Ribonuclease (RNase) R Digestion Assays

In the experiment involving actinomycin D, DLD-1 and LoVo cells were plated in 6-well dishes at a density of 5 × 10^4^ cells per well and allowed to adhere overnight. Subsequently, the cells were treated with 2 μg/mL of actinomycin D (Sigma-Aldrich, St. Louis, MO, USA) to inhibit transcription. Cells were collected at various time points: 0, 4, 8, 12, and 24 h post-treatment. For the RNase R digestion analysis, a total of 5 μg of RNA was incubated under two temperature conditions, i.e., −37 °C and 70 °C, each for 10 min, both in the presence and absence of 1.5 U/μg of RNase R (Geneseed, Guangzhou, China). The abundance of circ_RUSC2 and linear RUSC2 mRNA transcripts was quantified using quantitative reverse-transcription polymerase chain reaction (qRT-PCR).

### 4.6. Cytoplasmic and Nuclear RNA Fractionation

Cytoplasmic and nuclear fractions were separated using the PARIS Kit (Thermo Fisher Scientific, Waltham, MA, USA) according to the protocol of the manufacturer. Briefly, DLD-1 and LoVo cells (2 × 10^7^) were harvested and resuspended in cytoplasmic extraction buffer for 10 min and were centrifuged at 500× *g* for 5 min at 4 °C. The nuclei-containing pellet was redispersed in a nuclear extraction buffer solution and subsequently subjected to centrifugation at 500× *g* for a duration of 5 min. The expressions of circ_RUSC2, 18S rRNA (a cytoplasmic RNA marker), and U6 (a nuclear RNA marker) were examined in the cytoplasm and nucleus, and the percentage of each RNA molecule in the two fractions was calculated and compared.

### 4.7. RNA Pull-Down Assay

For the biotin-labeled miRNA pull-down experiment, the Pierce Magnetic RNA-Protein Pull-Down Kit (Thermo Fisher Scientific, Waltham, MA, USA) was utilized following the manufacturer’s instructions and established methodologies [51]. Briefly, DLD-1 and LoVo cells were inoculated with the biotin-conjugated miR-661 mimic (Bio-miR-661) or its corresponding negative control (Bio-miR-NC) using Lipofectamine 2000. After 48 h, the transfected cells were collected, lysed, and incubated with M-280 streptavidin-coated magnetic beads at 4 °C for 3 h. The beads were subsequently treated with an elution buffer and incubated at 37 °C for 30 min. RNA bound to the beads was extracted using the TRIzol reagent for a subsequent qRT-PCR analysis to evaluate the expression levels of circ_RUSC2 and TUSC2 mRNA. The sequences of the biotin-labeled miR-661 mimic and its negative control (GenePharma, Suzhou, China) are provided in Supplementary Table S2.

### 4.8. Cell Counting Kit-8 (CCK-8) Assay

Cells, plated at a density of 1 × 10^3^ per well in 96-well plates, were incubated for durations of 24, 48, 72, or 96 h. Subsequently, 10 μL of CCK-8 reagent (Thermo Fisher Scientific, Waltham, MA, USA) was dispensed into each well. Cell viability was then assessed using a microplate reader at 450 nm (Thermo Fisher Scientific, Waltham, MA, USA).

### 4.9. Scratch Wound Healing and Transwell Invasion Assays

Cells (2 × 10^5^) were plated and transfected in 6-well plates. A 1000 μL pipette tip was used to gently scratch a wound when the cells reached nearly 100% confluence. After 48 h, the proportion of the wound area that had been closed was observed and measured using Image J 1.46r.

A suspension of 5 × 10^4^ cells in 200 μL of serum-free medium was introduced into the upper compartments of Transwell inserts (Corning, Madison, NY, USA), which were pre-coated with Matrigel (BD Biosciences, San Jose, CA, USA). The lower compartments were filled with 600 μL of complete growth medium. Following a 24 h incubation period, non-migratory cells on the upper membrane surface were gently wiped away. Migrated cells adhering to the lower membrane surface were then fixed and stained using a 0.1% crystal violet solution for subsequent visualization and quantification.

### 4.10. Western Blot Analysis

Cell lysates were prepared from both parental and transfected cells, and protein concentrations were determined. Equal protein quantities (40 μg) were separated by electrophoresis on a 12% SDS–polyacrylamide gel and subsequently transferred onto membranes. Following a 3 h blocking step, the membranes were probed with primary antibodies targeting TUSC2 (1:1000 dilution, Abbkine, Wuhan, China), METTL3 (1:1000 dilution, ABclonal, Wuhan, China), and β-actin (1:1000 dilution, Proteintech, Wuhan, China) at 4 °C overnight. After washing, the membranes were incubated with a horseradish peroxidase (HRP)-conjugated rabbit anti-IgG secondary antibody (LI-COR Biosciences, Lincoln, NE, USA) at a 1:16,000 dilution in TBST for 1 h at room temperature. Protein signals were visualized and analyzed using the Odyssey (ODYSSEY 9120, California, USA) infrared imaging system (LI-COR Biosciences, Lincoln, NE, USA).

### 4.11. Immunohistochemistry

Tissue specimens were sliced into 4 μm thick sections and mounted onto slides. The sections were then incubated with a primary antibody targeting TUSC2 (1:100 dilution, Abbkine, Wuhan, China) overnight at 4 °C in a humidified chamber. After incubation, the slides were rinsed three times with PBS and subsequently treated with an HRP-conjugated secondary antibody for 1 h at 37 °C. Following DAB chromogenic development, TUSC2-positive cells were quantified under a light microscope (Nikon, Tokyo, Japan).

### 4.12. Statistical Analysis

Statistical evaluations were conducted employing SPSS version 20.0 software (IBM SPSS, Chicago, IL, USA) alongside GraphPad Prism version 8.0 (GraphPad Software, CA, USA). The data were presented as the mean accompanied by the standard deviation (SD). Comparisons between two groups were facilitated using Student’s *t*-test. For comparisons involving multiple groups, a one-way ANOVA was utilized. *T*-tests were applied to analyze the clinicopathological data. Statistical significance was considered when *p* < 0.05.

## 5. Conclusions

In conclusion, circ_RUSC2 and TUSC2 exhibited a low expression, while miR-661 exhibited a high expression in both CRC tissues and cells. These alterations correlated with advanced CRC stages, lymphatic and distant metastases, and poor differentiation. Circ_RUSC2 inhibited CRC cell proliferation, invasion, and migration, possibly through the absorption of miR-661 to release its target TUSC2. Circ_RUSC2 might become more stable through m6A methylation regulated by METTL3. This is the first study to highlight the tumor-suppressive role of circ_RUSC2 in CRC. Our results suggest that the circ_RUSC2/miR-661/TUSC2 axis represents a novel regulatory mechanism and could serve as a promising target for CRC diagnosis and therapy.

## Data Availability

The data in this article are available in the text and can be requested from the corresponding author upon a valid request.

## Supplementary Materials

The following supporting information can be downloaded at: https://www.mdpi.com/article/10.3390/ijms26072937/s1.
