# Supplementary material for: Circ_RUSC2 Sequesters miR-661 and Elevates TUSC2 Expression to Suppress Colorectal Cancer Progression
Source: Int J Mol Sci. 2025 Mar 24;26(7):2937. doi: 10.3390/ijms26072937 (PMC11989122; doi:10.3390/ijms26072937)
Supplement: Supplementary file 1 [file ijms-26-02937-s001.zip › ijms-3516588-supplementary.pdf]

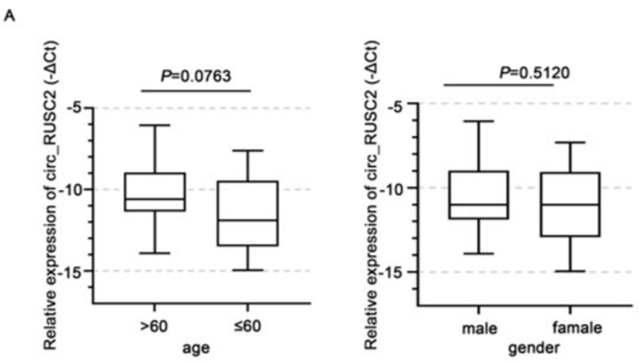

Figure S1 Analysis of clinicopathologic significance between relative expression of circ_RUSC2 and age (P = 0.0763), gender (P = 0.512) (P = 0.2879). Data were indicated as mean ± SD from 3 independent experiments.

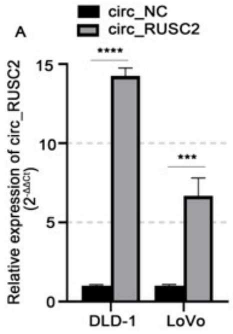

Figure S2 DLD-1 and LoVo cells were transfected with circ_NC or circ_RUSC2, the transfection efficiency was determined by qRT-PCR.

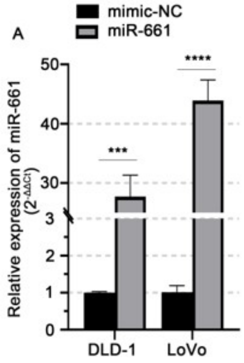

Figure S3 DLD-1 and LoVo cells were transfected with mimic-NC or miR-661, the transfection efficiency was determined by qRT-PCR.

Supplementary Table S1 Sequences of circ_RUSC2, miR-661 mimic and inhibitor, si-TUSC2 and si-METTL3

Name	Sequences
miR-661 mimic	Sense 5'-UGCCUGGGUCUCUGGCCUGCGCGU-3'
	Antisense 5'-GCGCAGGCCAGAGACCCAGGCAUU-3'
mimic-NC	Sense 5'-UUCUCCGAACGUGUCACGUTT-3'
	Antisense 5'-ACGUGACACGUUCGGAGAATT-3'
miR-661 inhibitor	5′-ACGCGCAGGCCAGAGACCCAGGCA-3′
inhibitor-NC	5′-CAGUACUUUUGUGUAGUACAA-3′
si-TUSC2-2	Sense 5'-GGAGACAAUCGUCACCAAGTT-3'
	Antisense 5'-CUUGGUGACGAUUGUCUCCTT-3'
si-METTL3	Sense 5'-GGACUGCGAUGUGAUUGUATT-3'
	Antisense 5'-UACAAUCACAUCGCAGUCCTT-3'
si-NC	Sense 5'-UUCUCCGAACGUGUCACGUTT-3'
	Antisense 5'-ACGUGACACGUUCGGAGAATT-3'

Supplementary Table S2 Primer sequences of circ_RUSC2, miR-661, TUSC2, RUSC2 and related internal controls

Name	Primer sequences
circ_RUSC2	Forward 5′-CCAGCCAACAGCCATACC-3′
	Reverse 5′-AGGAGAGCTTGGCACCGT-3′
miR-661	Forward 5′-TGCCTGGGTCTCTGGCCT-3′
	Reverse 5′-CCAGTGCAGGGTCCGAGGT-3′
TUSC2	Forward 5′-GGAGCTGAGCAAGCTTTGGT-3′
	Reverse 5′-CCGCTTCTGCCCGTTCTT-3′
RUSC2	Forward 5′-ATCGGGCAGCGTAGAACAT-3′
	Reverse 5′- CGAGGATGAAGGCGTTGAAG-3′
GAPDH	Forward 5′- CATGAGAAGTATGACAACAGCCT-3′
	Reverse 5′-AGTCCTTCCACGATACCAAAGT-3′

U6	Forward 5′-CGCTTCGGCAGCACATATACTA-3′
	Reverse 5′-GGAACGCTTCACGAATTTGC-3′
18S rRNA	Forward 5′-CCTCCAGGAGTCAATCCGATAC-3′
	Reverse 5′-CCATAAGCCGTTGGAGGAAA-3′
